# 

**Published:** 1887-01-22

**Authors:** 


					\jr(\
\KT ' /
? No. 17, Vol. I.
January 22nd, 1887.
PRICE ONE PENNY. 0
Per Annum, 6s. 6d.,post free.
Monthly Parts, 6d.; post free, 7?d.
Ditto per Ann., 7s. 6d., post free.
ait Institution, jfamilg, antr $arocf)taI journal of
hospitals, &s?Iums, $t all Agencies for tf)e Care of tfje 5>icft, Criticism & jjetos.

				

